# Biallelic variants in *CCN2* underlie an autosomal recessive kyphomelic dysplasia

**DOI:** 10.1038/s41431-024-01725-5

**Published:** 2024-11-06

**Authors:** Swati Singh, Sumita Danda, Neetu Sharma, Hitesh Shah, Vrisha Madhuri, Tariq Altaf Mir, Nadia Zipporah Padala, Raghavender Medishetti, Alka Ekbote, Gandham SriLakshmi Bhavani, Aarti Sevilimedu, Katta M. Girisha

**Affiliations:** 1 Department of Medical Genetics, Kasturba Medical College, Manipal, Manipal Academy of Higher Education, Manipal, India; 2 Department of Medical Genetics, Christian Medical College and Hospital, Vellore, Tamil Nadu India; 3 Centre for Innovation in Molecular and Pharmaceutical Sciences, Dr. Reddy’s Institute of Life Sciences, University of Hyderabad Campus, Gachibowli, Hyderabad, Telangana India; 4 Department of Pediatric Orthopedics, Kasturba Medical College, Manipal, Manipal Academy of Higher Education, Manipal, India; 5 Department of Pediatric Orthopedics, Christian Medical College and Hospital, Vellore, Tamil Nadu India; 6 Center for Rare Disease Models, Dr. Reddy’s Institute of Life Sciences, University of Hyderabad Campus, Gachibowli, Hyderabad, Telangana India; 7 Department of Genetics, College of Medicine and Health Sciences, Sultan Qaboos University, Muscat, Sultanate of Oman

**Keywords:** Genetics, Diseases

## Abstract

Kyphomelic dysplasia is a rare heterogenous group of skeletal dysplasia, characterized by bowing of the limbs, severely affecting femora with distinct facial features. Despite its first description nearly four decades ago, the precise molecular basis of this condition remained elusive until the recent discovery of de novo variants in the KIF5B-related kyphomelic dysplasia. We ascertained two unrelated consanguineous families with kyphomelic dysplasia. They had six affected offsprings and we performed a detailed clinical evaluation, skeletal survey, and exome sequencing in three probands. All the probands had short stature, cleft palate, and micro-retrognathia. Radiographs revealed kyphomelic femora, bowing of long bones, radial head dislocations and mild platyspondyly. We noted two novel homozygous variants in *CCN2* as possible candidates that segregated with the phenotype in the families: a missense variant c.443G>A; p.(Cys148Tyr) in exon 3 and a frameshift variant, c.779_786del; p.(Pro260LeufsTer7) in exon 5. *CCN2* is crucial for proliferation and differentiation of chondrocytes. Earlier studies have shown that *Ccn2*-deficient mice exhibit twisted limbs, short and kinked sterna, broad vertebrae, domed cranial vault, shorter mandibles, and cleft palate. We studied the impact of *CCN2* knockout in zebrafish models via CRISPR-Cas9 gene editing. F0 knockouts of *ccn2a* in zebrafish showed altered body curvature, impaired cartilage formation in craniofacial region and either bent or missing tails. Our observations in humans and zebrafish combined with previously described skeletal phenotype of *Ccn2* knock out mice, confirm that biallelic loss of function variants in *CCN2* result in an autosomal recessive kyphomelic dysplasia.

## Introduction

Kyphomelic dysplasia represents a heterogeneous group of rare genetic skeletal disorders, characterized by incurvation of limbs primarily affecting the femora, along with short stature, short and wide iliac wings, horizontal acetabular roof, platyspondyly, metaphyseal flaring and distinctive facial features that include prominent forehead, micrognathia, microstomia, cleft palate and low set ears [1–4]. The term “kypho” originates from ancient Greek word “kyphos” meaning “bent”, while “melia” refers to “limb”. The term ‘kyphomelia’ was first used to describe a skeletal dysplasia by MacLean in 1983 [1], noting an infant with broad and severely angulated short femora, congenital bowing of other long bones, narrow thorax, platyspondyly, micrognathia, and skin dimples while also comparing the clinical findings in four patients reported earlier. ‘Kyphomelic dysplasia’ was used to contrast with phenotype from ‘campomelic dysplasia’ that has less acute bending of femora.

In the recent nosology of genetic skeletal disorders [5], kyphomelic dysplasia is categorized within the bent bone dysplasia group among other entities namely, Campomelic dysplasia (OMIM# 114290), Cumming syndrome (OMIM# 211890), and Stuve–Wiedemann syndrome (OMIM# 601559), Kyphomelic dysplasia with facial dysmorphism, KIF5B related (OMIM# 614592), Bent bone dysplasia, FGFR2 related (OMIM# 614592), Bent bone dysplasia, LAMA5 related (OMIM# 620076). Kyphomelic dysplasia can be misdiagnosed as campomelic dysplasia, where mild bowing of the femur and severe anterior bowing of the tibia are often observed or any other disorder listed here [6, 7]. Conventionally, it was considered an autosomal recessive condition [2, 8]. Recently, heterozygous de novo variants in *KIF5B* have been found to be associated with a kyphomelic dysplasia [4], while several bent bone dysplasias do not have a known genetic basis.

Cellular communication network factor 2 (CCN2), also referred to as connective tissue growth factor (CTGF), facilitates interactions with growth factors, cell surface proteins and extracellular matrix components. It is a matricellular protein crucial for skeletal growth and development, playing a vital role in regulating the differentiation and function of osteoblasts and osteoclasts [9, 10]. In vitro studies on *CCN2* demonstrated that it promotes DNA synthesis in chondrocytes [11]. Investigations on *Ccn2* deficient mice showed broader vertebrae, shortened and kinked sterna, along with bending in the radius, ulna, tibia, and fibula. Additionally, they exhibit craniofacial abnormalities including a distorted ethmoid bone, a domed cranial vault, shortened mandibles and secondary cleft palate [12]. Histologically, osteochondrogenesis, osteogenesis and osteoclastogenesis appear to be disturbed [12, 13].

We evaluated three probands from two unrelated consanguineous families with bowed long bones, particularly severe in femora, and identified biallelic disease-causing variants in *CCN2*, segregating in a recessive manner. We investigated consequences of loss of function of *CCN2* activity in vivo in zebrafish models.

## Methods

### Participants

Three individuals with kyphomelic dysplasia, from two unrelated families of different ethnicities were recruited in the study. Comprehensive medical history, clinical assessments, and radiological findings of all affected individuals were documented. Written informed consents were obtained from the affected individuals and their family members. The study has received the approvals from the institutional ethics committees of Kasturba Medical College and Hospital, Manipal and Christian Medical College, Vellore.

### Genetic testing

Exome sequencing was performed for all the three affected individuals with kyphomelic dysplasia as described in supplementary information. Filtered variants were further analyzed using in silico pathogenicity prediction tools (such as CADD Phred, MCAP, Mutation Taster, REVEL, SIFT Indel, AlphaMissense) to assess their potential impact. Conservation analysis using the Clustal Omega tool [14], was performed to assess the conservation of the amino acid residue across species. The allele frequency of the identified rare variant was estimated from gnomAD (V3.1.2), and our in-house data of 3188 exomes.

Sanger sequencing was performed to validate and segregate identified candidate variants in the proband and their family members. The variants were described according to Human Genome Variation Society (HGVS) nomenclature, with NCBI reference sequences (NM_001901.4, NP_001892.2). Both variants were submitted to the Leiden Open Variation Database (LOVD) database (variant ID: 0000972076; 0000972075). To visualize disulfide bridge between cysteine 148 and a nearby cysteine residue, computed structure models from AlphaFold DB with The PyMOL Molecular Graphics System, Version 3.0 Schrödinger, LLC was utilized.

### Generation of knockout zebrafish models

CRISPR/Cas9 mediated gene editing was used for generation of *ccn2a* F0 knockout in zebrafish. In the F0 knockout studies, a multi guide-RNA mix and Cas9 protein are injected into one-cell stage embryos at a much higher concentration than used typically for line generation. This results in stable biallelic edits in the target gene in a subset of injectants, revealing the loss of function phenotype in the injectant (F0) generation. The rationale is similar to that of using morpholinos to create a transient knockdown in the injectants, however the edits induced and the resulting phenotype are stable, and may be studied in (F0) adults as well. Additionally, since phenotypes are studied in the injectants (crispants), compensation via genetic adaptation does not arise, as may be the case in a stable knockout studied in the F2 generation. However, crispants are genotypically mosaic, and the fraction of crispants with complete biallelic loss of function may vary in each set of injectants. Benchling software was used to choose target region of *ccn2a*. A non-target sequence not present in the zebrafish genome was chosen as a control (NT) [15]. Single guide RNA (sgRNAs) were synthesized, as described in the studies by Medishetti et al. and Sorlien et al. [16, 17]. Phenotypic characterization of the *ccn2a* F0 knockout zebrafish was performed as described earlier [18]. Subsequently, Alcian blue staining was performed [19]. Quantitative real-time qPCR analysis was done for investigating gene expressions of *ccn2a, rac1a, rhoAa, col2a1a, sp7, runx2a and gapdh* using Ct method (∆∆Ct) (methods are described in greater detail in the supplementary information).

## Results

### Clinical findings

**Fig. 1:**
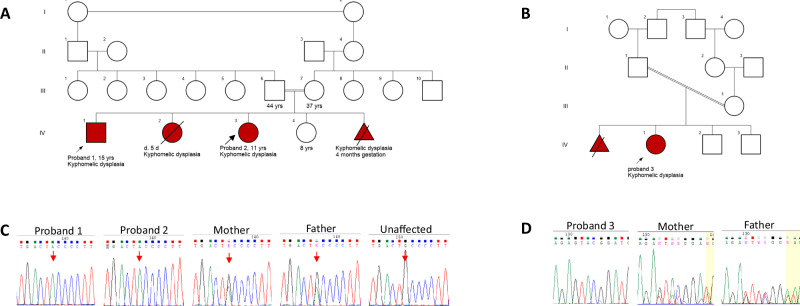
Pedigrees and Sanger chromatograms. **A** Pedigree of the family 1 and **B** family 2 depicting consanguinity and affected probands with kyphomelic dysplasia. **C** Sanger chromatograms in family 1 show the variant c.448G>A in homozygous state in both affected siblings, heterozygous state in their parents and absent in their unaffected sibling. **D** Chromatograms of family 2 confirm the variant c.779_786delG>A in homozygous state in proband 3 and heterozygous state in her parents.

**Fig. 2:**
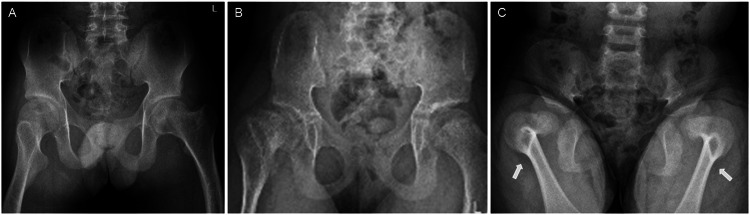
Kyphomelic femora in the participants. Radiographs of pelvis [proband 1 at 15 years (**A**), proband 2 at 11 years (**B**) and proband 3 at 3 years of age (**C**)] show short and broad pelvis, coxa vara and reduced hip joint space in all. Kyphotic femora are seen in all of them but is more prominent (arrows) in the younger proband 3 (**C**).

**Fig. 3:**
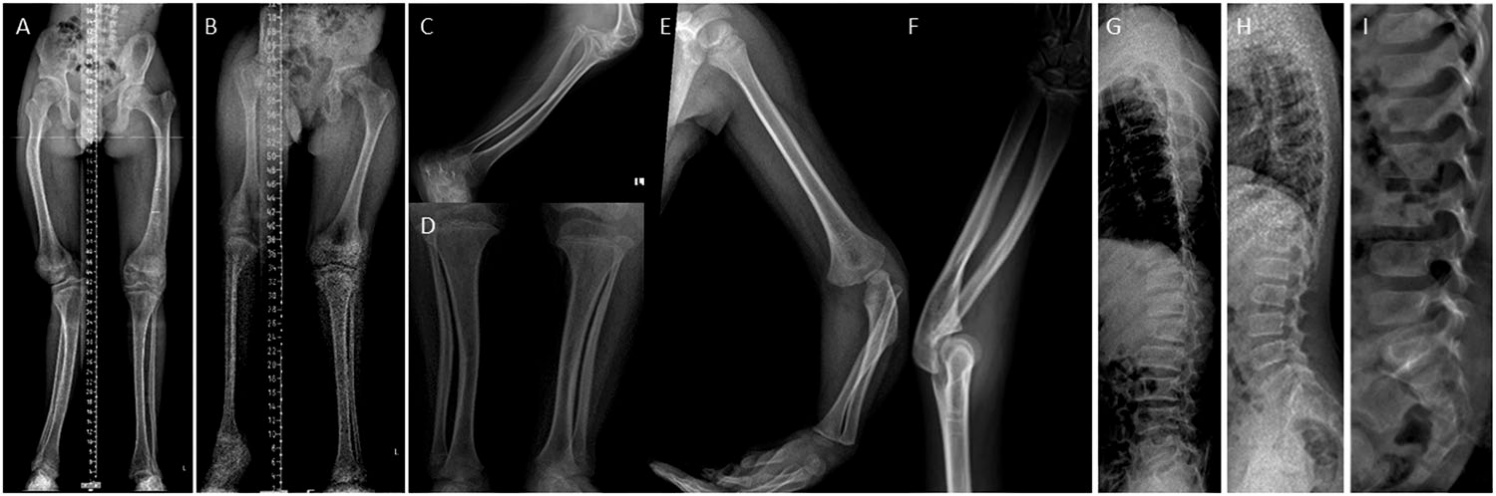
Bowing of long bones in the affected individuals. Radiographs of limbs of probands: proband 1 at 15 years (**A**), proband 2 at 11 years (**B**) and proband 3 at 3 years of age (**C**–**F**) show bowing of long bones. Bowing of femur (**A** and **B**), bowing of the tibia and fibula (**A**, **C**, **D**) can be observed. Irregular epiphyses and metaphyses along with flaring of metaphyses at the knee joint can be noted (**D**). Variable platyspondyly can be observed in all (**G**–**I**).

Three probands (P1 and P2 from family 1 and P3 from family 2) (Fig. 1) were noted to have short long bones antenatally at the fifth month of gestation. Cleft palate was observed in all of them (cleft uvula in P2). They demonstrated short stature, facial dysmorphism, and kyphomelic skeletal dysplasia. Facial abnormalities include bitemporal narrowing, posteriorly placed ears, deviated nasal septum, micrognathia, microstomia, retrognathia, and crowded teeth (Supplementary Fig. 1). Major radiographic findings consisted of kyphomelic femora and bowed tibia, fibula, radius and ulna. Additionally, radial head dislocation, scoliosis, mild platyspondyly, broad and short pelvis with horizontal acetabulum and reduced joint space, coxa vara, patellar dislocation and irregularities of the knee epiphyses and metaphyses were noted (Figs. 2, 3, Supplementary Figs. 2–6). The phenotypic manifestations in family 1 were milder compared to those in family 2. Detailed clinical and radiological findings are provided in the supplementary information.

### Molecular findings

Exome sequencing in three probands from the two unrelated families did not reveal any variants that can support the diagnosis of known bone diseases with bent long bones. In Family 1, a shared biallelic missense variant, c.443G>A; p.(Cys148Tyr), located in exon 3 of the *CCN2* (NM_001901.4; NP_001892.2), was identified through duo exome sequencing. This variant is absent in gnomAD (V3.1.2) and our in-house data of 3188 exomes. It is predicted to substitute a cysteine residue with tyrosine in the von Willebrand factor type C (VWC) domain of the CCN2 protein. Multiple sequence analysis performed using the Clustal Omega tool revealed conservation of the cysteine residue across several vertebrate species (Supplementary Fig. 7). In silico pathogenicity prediction tools, including CADD phred: 31.00 and REVEL: 0.967, predicted the variant to be disease-causing. Additionally, the AlphaMissense score for the variant p.(Cys148Tyr) is 0.996, further indicating its potential pathogenicity. In silico mutagenesis analysis revealed a disulfide bridge formed between cysteine residue at position 148 and the nearby cysteine at position 166 (Supplementary Fig. 8), is disrupted due to the substitution. The variants are present in heterozygous state in the parents and absent in the unaffected sibling.

In family 2, we identified a homozygous frameshift variant, c.779_786del; p.(Pro260LeufsTer7) that was absent from the public and in-house datasets, located in exon 5 of *CCN2*. Exon 5 of *CCN2* encodes for carboxyterminal domain, which is critical for interaction with cell surface integrins [20]. Sanger sequencing confirmed the heterozygous status of the parents. Detailed clinical, radiographic, and molecular findings of probands are summarized in Table 1.

**Table 1:** Summary of clinical and genetic findings in individuals with CCN2 variants and kyphomelic dysplasia.

Family ID	Family 1		Family 2
Subject ID	P1	P2	P3
General characteristics			
Age at last evaluation	15 years	11 years	11 years
Gender	Male	Female	Female
Consanguinity	+	+	+
Anthropometry			
Height in cm (SD)	153 cm (−2.03)	118 cm (−3.6)	76 cm (−5.5) at 3.5 years
Weight in kg (SD)	41 kg (−1.91)	25 kg (−2.18)	14 kg (+0.06) at 3.5 years
Head circumference in cm (SD)	52.5 cm (−1.61)	50.5 cm (−1.71)	49 cm (−2.94)
Clinical findings			
Short stature	+	+	+
Microcephaly	–	–	+
Bitemporal narrowing	+	+	+
Posteriorly placed ears	+	+	+
Deviated nasal septum	+	-	+
Micrognathia	+	+	+
Retrognathia	+	+	+
Microstomia	+	+	+
Cleft palate/ bifid uvula	+	+	+
Crowded teeth	+	+	+
Muscle wasting	+	+	+
Skeletal findings			
Scoliosis	+	+	–
Platyspondyly	+	+	+
Short and broad pelvis	+	+	–
Coxa vara	+	+	+
Epiphyseal dysplasia	+	+	+
Metaphyseal dysplasia	+	+	+
Elbow joint dislocation	+	+	+
Bent radius and ulna	+	+	+
Limited elbow extension/joint mobility	-	+	+
Femoral bending (kyphomelia)	+	+	+
Bowing of tibia and fibula	+	-	+
Mobile patella	+	–	+
Windswept deformity	+	–	–
Genu valgum	–	+	+
Limited knee flexion	+	+	+
Bilateral pes planus	+	–	–
Pes cavus	–	–	+
Broad great toe	+	+	+
Molecular findings in *CCN2*			
Nucleotide change (NM_001901.4)	c.443G>A	c.443G>A	c.779_786del
Amino acid change (NP_001892.2)	p.(Cys148Tyr)	p.(Cys148Tyr)	p.(Pro260LeufsTer7)
Location	Exon 3	Exon 3	Exon 5
Zygosity	Homozygous	Homozygous	Homozygous
Parental status	Heterozygous	Heterozygous	Heterozygous
Variant effect	Missense	Missense	Frameshift

+ Present, − Absent

### Functional studies on zebrafish

**Fig. 4:**
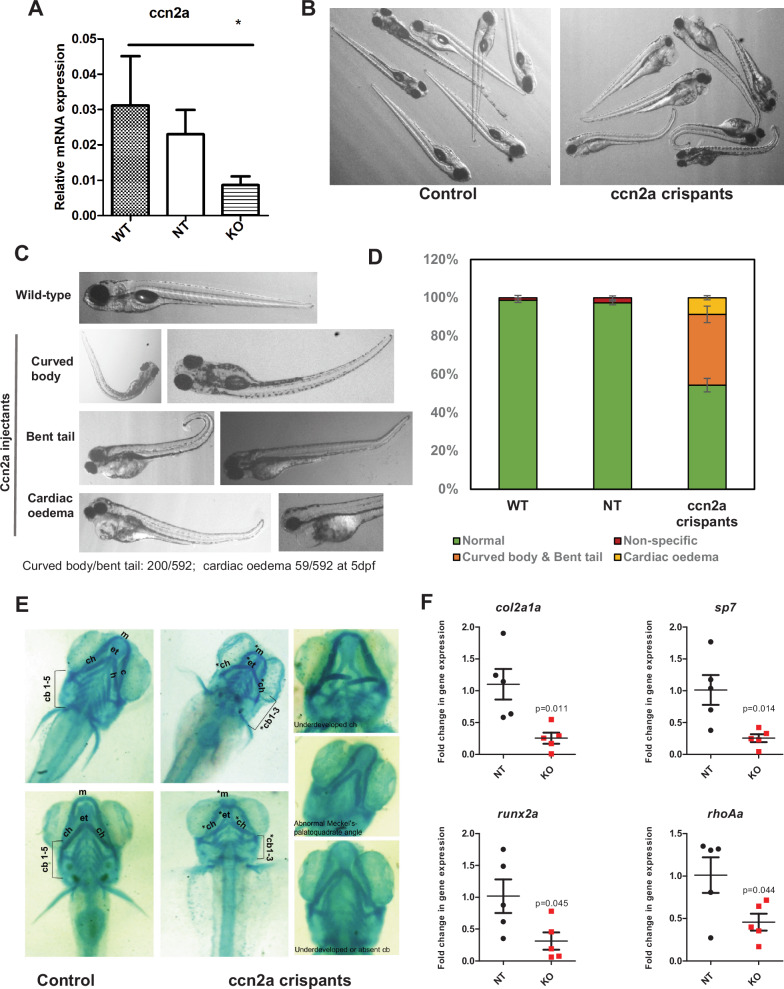
Phenotypes in the *ccn2a* FO knockout zebrafish. **A** Relative expression levels of the ccn2a mRNA in the controls (NT) and injectants (crispants). **B** Bright field image of a group of *ccn2a* crispants and matched control injectants from the same experiment to illustrate the extent of physical phenotypes. **C** Representative images of observed phenotypes as labeled, among the *ccn2a* crispants. **D** Quantification of the number of larvae showing the indicated phenotypes in each group. **E** Alcian blue stained images of wildtype and representative examples of *ccn2a* crispants, showing cartilage deformities. Cartilage elements labeled: m: Meckel’s cartilage; ch: ceratohyal; cb: ceratobranchial pairs. **F** Fold change in mRNA expression of select markers such as *col2a1a* (chondrocyte), *sp7* (osteoblast), *runx2a* (early osteoblast) and rho1a (palatogenesis) in the *ccn2a* crispants as compared to the controls at 5dpf. Results from at least 5 independent experiments (pooled larvae, 30–50/experiment) are quantified.

A significant decrease in *ccn2a* mRNA was observed in the crispants as compared to the NT and WT controls, thus confirming *ccn2a* editing (Fig. 4A). The *ccn2a* crispants showed abnormal body curvature and bent tail suggesting defects in early skeletal development (Fig. 4B). A small but significant fraction of crispants showed severe cardiac edema. The number of crispants showing these phenotypes was quantified in each experiment, and representative images and quantification are shown (Fig. 4C, D). The ccn2a crispants showed substantial defects in cartilage formation in the craniofacial region as seen by Alcian blue staining at 6.5 days post-fertilization (Fig. 4E). They had underdeveloped ceratohyal arches, bent or missing ceratobranchial arches and misshapen Meckel cartilage.

To further confirm a role for *ccn2a* in early cartilage and bone formation, we examined the levels of established skeletal marker genes such as *col2a1a* (chondrocyte marker), *rac1a* and *rhoAa* (palatogenesis markers), *sp7* and *runx2a* (osteoblast markers). The *ccn2a* crispants showed a significant decline in the levels of several of these markers, as is expected from the phenotype (Fig. 4F and Supplementary Fig. 9C).

The crispants also showed poor survival beyond seven days post-fertilization (dpf) and few survived to adulthood. These adult crispants (F0 KO) showed defects in mineralization and bone structure in specific locations with known endochondral ossification (Supplementary Fig. 10C) such as missing structures in the tail region (hypural bones) and abnormal trunk curvature (Supplementary Fig. 10A, B). The level of knockdown of *ccn2a* in these adults was confirmed by measuring the mRNA level from trunk tissue and was found to be significantly reduced (Supplementary Fig. 10C).

## Discussion

We describe an autosomal recessive kyphomelic dysplasia in multiple affected individuals. We identified two homozygous variants (missense and frameshift) in *CCN2*, which encodes a protein involved in proliferation and differentiation of chondrocytes. Further, we investigated the consequences of loss of function of *CCN2* in zebrafish models.

Affected individuals reported here manifested with sharp femoral angulation spontaneously alleviating with age and minor facial dysmorphism. Their clinical and radiological manifestations were consistent with what were previously reported in kyphomelic dysplasia [1, 6–8]. The manifestations were much milder in Family 1 than in Family 2, probably representing different phenotypic consequences between a missense and a truncating variant.

Although numerous reports have described about 23 patients with kyphomelic dysplasia, none of these have provided molecular etiology of the disorder [6, 21–23]. It is possible that some of these might represent other genetic disorders with bent long bones and even have a non-genetic etiology. Nevertheless, kyphomelic dysplasia is typically regarded as an autosomal recessive condition in the reported cases [2, 8]. In a recent study Itai et al., described de novo heterozygous variants in *KIF5B* leading to kyphomelic dysplasia in four individuals [4]. Affected individuals had short stature, bowing of limbs and facial dysmorphism including bitemporal constriction, arched eyebrow, hypertelorism, proptosis, ptosis, midface hypoplasia, micrognathia and cleft palate. CCN2 is a secreted protein, and its molecular function is predominantly that of a growth factor, acting extracellularly via interactions with extracellular matrix (ECM) proteins, growth factors and their receptors (BMPs, FGF2) and other CCNs, promoting proliferation and differentiation of chondrocytes, osteoblasts and osteoclasts [10, 11, 24]. KIF5B is a Kinesin family motor protein, an intracellular protein required for organelle transport, especially mitochondria and lysosomes, nuclear positioning and plays an important role in the control of autophagy. Although detailed mechanistic studies are unavailable, it appears likely that dysregulated autophagy in kif5b loss of function results in a loss of hypertrophic chondrocytes, and significantly impairs the extensive cartilage remodeling that occurs during early skeletogenesis [25]. These two proteins, one acting outside the cell and one predominantly inside, appear to play crucial but distinct roles in chondrogenesis.

CCN2 is a multifunctional protein spanning 349 amino acids, belonging to the cysteine rich CCN protein family, which shows conservation of all cysteine residues [26]. It comprises of four modules, namely: IGF (insulin like growth factor)-binding protein-like (IGFBP), von Willebrand factor type C (VWC), thrombospondin type 1 repeat (TSP1) and C-terminal cysteine knot (CT) [27]. Each of these modules serve distinct functions. *CCN2* gene has five exons and the variants identified in the study reside in exons 3 and 5 affecting VWC and CT domains of the protein (Supplementary Fig. 7).

*Ccn2* deficient mice exhibit perinatal lethality due to respiratory failure primarily attributed to short and bent sterna and kinked ribs. These findings closely mirrored the clinical manifestations observed in our study [9, 12, 13, 28, 29]. In studies in knockout mice, absence of CCN2 is also reported to inhibit palatal shelf elevation from the vertical to horizontal position thus demonstrating its importance in mammalian palatogenesis. This can be correlated with the occurrence of cleft palate in humans, which is evident in all probands in the study [30]. Mice homozygous for deletion of *Ctgf* gene die soon after birth.

Given the high conservation of fundamental signaling pathways and cellular processes involved in skeletal development from fish to humans, zebrafish serve as valuable models for studying skeletal disorders [28, 31]. Numerous human skeletal disorders have been successfully replicated in zebrafish models [32]. In zebrafish, *ccn2a* has been shown to play an important role in heart regeneration after cardiac injury [33]. It has also been reported to have regenerative activity and is required for spinal cord regeneration, pro-regenerative activity of *ccn2a* maps to its C-terminal domains [33, 34], however its function in early skeletal development in zebrafish has not yet been studied. The previous reports using the *ccn2a−/−* line did not report any major developmental or skeletal phenotypes [35]. We believe this could be due to genetic compensation mechanisms which may arise due to the severe defects associated with loss of *ccn2a*, as has been reported previously for select genes [36], especially given that significant compensatory upregulation of *ccn2b* was observed in the *ccn2a*−/− line (which we did not observe in the *ccn2a* crispants (Supplementary Fig. 9B). To circumvent these issues, we have therefore used an effective F0 knockout strategy described by [37], to create a loss of *ccn2a* function and study the impact during larval stages on cartilage formation and subsequently mineralization. This method has been reported to recapitulate knockout phenotypes in more than 90% of F0 embryos (crispants), with persistence well into adulthood.

In *ccn2a* crispants, we observed skeletal developmental abnormalities similar to the phenotypes observed in patients with *CCN2* variants which led to skeletal dysplasia and cleft palate. A significant decrease in osteogenic markers like *col2a1a*, *sp7*, and *runx2* was noted during zebrafish development. This decline may be attributed to reduced CCN2 expression, which plays a pivotal role in inducing these markers through various pathways [13]. The comparison of phenotypes observed among zebrafish, mice and humans are described in Table 2.

**Table 2:** Comparison of phenotypes observed in zebrafish, mice and human due to loss of function of *CCN2* gene.

Phenotypes observed due to loss of function of *CCN2*	*ccn2a* knockout zebrafish	*Ccn2* deficient mice	Individuals with biallelic *CCN2* variants
Craniofacial abnormalities	Underdeveloped ceratohyal arches, bent ceratobranchial arches and misshapen Meckel’s cartilage	Shortened mandibles, deformed Meckel’s cartilage and ethmoid bones, domed cranial vault, and secondary cleft palate	Bitemporal narrowing, posteriorly placed ears, deviated nasal septum, short/bifid uvula, crowded teeth, micrognathia, microstomia and retrognathia
Vertebral/ ribs abnormalities	Altered body curvature	Short and bent sterna and kinked ribs	Platyspondyly, mild scoliosis and irregularities at vertebral ends
Upper limb deformities	Not determined	Bends in radius, ulna	Radial head dislocation and bent radius and ulna
Lower limb deformities	Bent or missing tail	Curved tibia and fibula	Kyphomelic femur, bent tibia and fibula, broad great toes, genu valgum and windswept deformity
Epi-metaphyseal dysplasia	Not determined	Not determined	Irregular epiphyses and irregular and flared metaphyses
References	Present study	Ivkovic et al., 2003	Present study

We acknowledge certain limitations in our study. We are unable to provide cellular effects of CCN2 variants in these patients, such as immunocytofluorescence and western blot analyses. These analyses would have allowed us to examine expression patterns of CCN2 variants and osteogenic markers compared to control samples. We report only on F0 knockouts in this study, as creating zebrafish lines expressing mutant Ccn2a proteins would be technically challenging, time-consuming and financially demanding, and beyond the scope of our current expertise.

In summary, we present two unrelated families with multiple affected individuals with an autosomal recessive kyphomelic dysplasia, resulting from likely loss of function of the *CCN2*. The mice knockouts have already been described to have bone dysplasia akin to the human phenotype described here. We also show zebrafish knockouts for *ccn2a* show skeletal abnormalities. However, investigation of additional patients and cellular studies are necessary to establish the gene-disease relationship.

## Web resources

PRIMER 3v.4.1.0, http://primer3.ut.ee/

Ensembl, https://asia.ensembl.org/index.html

Mutation Taster, http://www.mutationtaster.org/

CADD Phred, https://cadd.gs.washington.edu/

MCAP, http://bejerano.stanford.edu/mcap/

REVEL, https://genome.ucsc.edu/cgi-bin/hgTrackUi?db=hg19&g=revel

SIFT Indel, https://sift.bii.a-star.edu.sg/www/SIFT_indels2.html

Clustal Omega, https://www.ebi.ac.uk/jdispatcher/msa/clustalo

PyMOL, (https://www.pymol.org/)

AlphaMissense: https://alphamissense.hegelab.org/search

Online Mendelian Inheritance in Man (OMIM): https://www.omim.org/

gnomAD, https://gnomad.broadinstitute.org/

HPO, https://hpo.jax.org/app/

LOVD, https://www.lovd.nl/

ANNOVAR, http://annovar.openbioinformatics.org/

## Supplementary information

Supplementary information

Supplementary material

## Data Availability

The data that support the findings of this study are available from the corresponding author upon reasonable request.
