# Supplementary material for: Biallelic variants in CCN2 underlie an autosomal recessive kyphomelic dysplasia
Source: Eur J Hum Genet. 2024 Nov 6;33(1):30–7. doi: 10.1038/s41431-024-01725-5 (PMC11711675; doi:10.1038/s41431-024-01725-5)
Supplement: Supplementary file 1 — Supplementary information [file 41431_2024_1725_MOESM1_ESM.docx]

**Supplementary information for**

**Biallelic Variants in *CCN2* Underlie an Autosomal Recessive Kyphomelic Dysplasia**

Swati Singh^1^, Sumita Danda^2^, Neetu Sharma^3^, Hitesh Shah^4^, Vrisha Madhuri^5^, Mir Tariq Altaf^5^, Nadia Zipporah Padala^3^, Raghavender Medishetti^3^, Alka Ekbote^2^, Gandham SriLakshmi Bhavani^1^, Aarti Sevilimedu^3,6^, Katta M Girisha^1,7^

^1^Department of Medical Genetics, Kasturba Medical College, Manipal, Manipal Academy of Higher Education, Manipal, India.

^2^Department of Medical Genetics, Christian Medical College and Hospital, Vellore, Tamil Nadu, India.

^3^ Centre for Innovation in Molecular and Pharmaceutical Sciences, Dr. Reddy’s Institute of Life Sciences, University of Hyderabad Campus, Gachibowli, Hyderabad, Telangana, India.

^4^Department of Pediatric Orthopedics, Kasturba Medical College, Manipal, Manipal Academy of Higher Education, Manipal, India.

^5^Department of Pediatric Orthopedics, Christian Medical College and Hospital, Vellore, Tamil Nadu, India.

^6^Center for Rare Disease Models, Dr. Reddy’s Institute of Life Sciences, University of Hyderabad Campus, Gachibowli, Hyderabad, Telangana, India.

^7^Department of Genetics, College of Medicine and Health Sciences, Sultan Qaboos University Muscat, Sultanate of Oman.

**Corresponding Author**

**Katta M. Girisha**

Professor

Department of Genetics,

College of Medicine and Health Sciences,

Sultan Qaboos University Muscat, Oman

and

Department of Medical Genetics,

Kasturba Medical College, Manipal,

Manipal Academy of Higher Education, Manipal,

Manipal, Karnataka, India

Contact number: +968 24143488

Email address: [girish.katta@manipal.edu](mailto:girish.katta@manipal.edu); [g.kumar@squ.edu.om](mailto:g.kumar@squ.edu.om)

**Methods**

**Genetic testing**

Peripheral blood (2-5 ml) was collected from the probands, siblings and their parents. The genomic DNA was isolated using QIAmp DNA Blood Mini kit (QIAGEN, Hilden, Germany) following the standard protocols. Exome sequencing was performed for all the three affected individuals with kyphomelic dysplasia. Exome sequencing utilized the Agilent sure select TWIST V6 exome capture kit (TWIST Biosciences, South San Francisco, California, USA) and run on HiSeq2000 platform (Illumina San Diego, CA, USA), achieving an average coverage of 100X, with >95% of bases covered at a minimum of 20X and 97% sensitivity. Raw data was retrieved in FASTQ format and aligned to the GRCh38 assembly using Burrows-Wheeler Aligner (v2.2.1) and our in-house pipeline based on the 'Genome Analysis Toolkit Best Practices' (1). Subsequently, the data underwent annotation by 'Annotate Variation (ANNOVAR)' (2), supplemented by our in-house scripts (3). Filtered variants were further analyzed using *in silico* pathogenicity prediction tools and conservation analysis tool (4) to assess their pathogenicity. The gnomAD (V3.1.2), and our in-house data were referred to estimate variants allele frequency.

Sanger sequencing was performed to validate and segregate identified candidate variants in the proband and their family members. Primers were designed using Primer3 software. The sequencing was carried out on an ABI 3500 Genetic Analyzer, according to the manufacturer's protocol.

**Design and synthesis of Single guide RNA (sgRNA), and microinjection**

The zebrafish genome contains two *CCN2* orthologs *ccn2a* and *ccn2b*, among which we chose to work with *ccn2a* for two primary reasons: a) *ccn2a* is phylogenetically closer to human *CCN2*, and b) *ccn2b* is not well expressed during early development (supplementary figure 11A-D, 9A) (5). In order to determine whether the loss of *ccn2a* function leads to impaired skeletal development, we created F0 knockouts of *ccn2a* in zebrafish as reported previously (6). We designed four guides targeting the *ccn2a* locus, spanning the entire coding sequence and tested these for efficacy of editing in vivo, by injecting the Cas9-sgRNA RNP (ribonucleoprotein) complexes into 1-cell stage embryos and performing genotyping at 24hpi (hours post injection) by HMA PCR. Two of the four guides (targeting exon 3 and 4) were chosen based on efficacy and used at 1ng guide RNA mix per embryo for all subsequent experiments (Supplementary Figure 12A and B). Two controls were used for each experiment, uninjected embryos (WT) and injections with a non- targeting guide RNA (NT) injected at the same amount as the ccn2a guides. Single guide RNA (sgRNA) template for each, was ordered as single stranded DNA with the addition of T7 promoter at 5’end and tail oilgo sequence at 3’end with the following final sequences:

sgRNA3: TAATACGACTCACTATAGGgactgcccaatgccccgcaGTTTTAGAGCTAGAA

sgRNA4: TAATACGACTCACTATAGggcgagactgcttctcaaggGTTTTAGAGCTAGAA

NT gRNA: TAATACGACTCACTATAGggggaggcgttcggccacagGTTTTAGAGCTAGAA

sgRNAs were synthesized, as described in the studies by Medishetti et al and Sorlien et al (7,8). Briefly, templates for sgRNA3 and sgRNA4 were obtained by carrying out overlap PCR using T7 promoter forward primer and tail oligo reverse primer. The product was ethanol precipitated and in vitro transcribed using HiScribe™ T7 RNA synthesis kit (E2040S, New England Biolabs, Massachusetts, USA) to get the individual sgRNAs. A ccn2a guide RNA mix (sg3+sg4) or NT gRNA gRNA was allowed to form a complex with Cas 9 protein by incubating at 37°C for 10 minutes. This ribonucleoprotein (RNP) complex was microinjected (effective concentration of guides 1 ng per embryo along with 400 ng of Cas 9 protein) in one cell stage embryos, followed by incubation of the embryos at 28ºC. The indels were visualized using the heteroduplex mobility assay (HMA) on 10% native PAGE after 24 hours post microinjection. Differential amplicon migration as compared to wild type control indicated presence of indels and confirmed effective editing in vivo. Phenotypes were observed and imaged at 5dpf (days post fertilization) using bright-field microscopy (Zeiss Stereo Discovery.V8) using 3.5x magnification and as per published methodology (9).

**Alcian blue staining**

At 6.5dpf, zebrafish larvae were fixed in 4% paraformaldehyde (PFA) overnight. Subsequently, Alcian blue staining was performed as per the method described in earlier publication (10). Briefly, fixed embryos were washed three times with phosphate-buffered saline (PBS) for 10 minutes and with 50% ethanol for 30 minutes. After ethanol wash, embryos were stained overnight at room temperature with 0.4% alcian blue prepared in 20mM MgCl_2_ and 70% ethanol. After staining, embryos were washed with 70% ethanol/10 mM MgCl_2_, 50% ethanol/10 mM MgCl_2_ and 25% ethanol, respectively. To reduce staining of soft tissues, embryos were bleached with 3% H_2_O_2_ and 2% KOH for 15 minutes. Embryos were washed with 25% glycerol and 0.25% KOH and stored in 50% glycerol in 0.25% KOH. Stained embryos were positioned in this storage solution in slits of the agarose mould and the head was photographed in a ventral–dorsal view. The defects in facial cartilage (Ceratobranchial pairs, Meckel's cartilage, Ceratohyal) were visualized and images were compared with the wild type and NT controls.

**Quantitative real-time qPCR analysis**

Total RNA was isolated using Trizol from 50 larvae at 5dpf for individual batches of injectants in n=5 experimental sets. cDNA was synthesized with 600 ng of total RNA using PrimeScript™ RT reagent Kit (RR037A-Takara Bio, Kusatsu, Japan) in a reaction volume of 10µl. qPCR was performed using TB Green mix on QuantStudio5 (Applied Biosystems, California, USA). Data were analyzed using the Ct method (∆∆Ct) and normalized to the RNAPD reference gene. 0.3µL of cDNA was used per reaction with zebra fish specific exonic primers for each of the genes studied that is *ccn2a, rac1a, rhoAa, col2a1a, sp7, runx2a and rnapd*. A fold change in gene expression was calculated for the nontargeting (NT) injectant controls and *ccn2a* guide injectants for each experiment. The fold change in gene expression from at least 5 independent experiments was plotted using Graph Pad Prism 5. The statistical significance was calculated using unpaired t-test.

**Results**

**Clinical description**

Family 1: We ascertained consanguineously married couple with five pregnancies. Two living children (proband 1 and 2) have a kyphomelic dysplasia. They lost an affected female child and an affected pregnancy was medically terminated. They have a healthy daughter too (Figure 1).

Proband 1 (P1) is a 15-years-old boy, with skeletal deformities. Antenatally, short long bones were noted at fifth month of gestation. He was delivered at full term through normal vaginal delivery with a birth weight of 2 kg (-2.5 SD). He cried immediately after birth and experienced no perinatal complications. Difficulty in feeding and regurgitation through the nose due to cleft palate was noted on sixth day of life. He attained head control at seven months, sat without support at two-years, stood at two-years-six-months and walked at three years. He frequently fell while walking. He had habitual dislocation of the left patella, for which he underwent surgery at nine years of age. A left knee lateral release (medial patellofemoral ligament), reconstruction with iliotibial banding and vastus medialis advancement was performed. Surgical correction for the cleft palate was carried out at 1.5 years of age. Currently, he is in ninth grade and exhibits good scholastic performance.

At 15 years of age, his height was 153 cm (-2.03SD), weight was 41 kg (-1.91 SD) and head circumference was 52.5 cm (-1.61 SD). A thorough clinical evaluation showed short stature, facial dysmorphism with bitemporal narrowing, posteriorly placed ears, deviated nasal septum, short uvula (operated), crowded teeth, micrognathia, microstomia and retrognathia. Additionally, bilateral muscle wasting, wind-swept deformity, mobile patella of the right knee, bilateral pes planus and broad great toes were observed (supplementary figure 1).

Radiographic findings suggest radial head dislocation of the right elbow, bent radius and ulna, mild scoliosis with platyspondyly and vertebral irregularities, short and broad pelvis with horizontal acetabulum and reduced joint space, coxa vara on the left side, bowing of femora and tibia, irregular epiphyses of the knee, and generalized irregularity and flaring of metaphyses (figure 2-3 and supplementary figure 2-6).

Proband 2 (P2) is a 11-years-old sister of P1 and has left genu valgum and difficulty in walking. She was born at full term via normal vaginal delivery, with a birth weight of 2.7 kgs (-1.38 SD). Similar to her elder sibling, antenatal sonographic examination showed shortening of long bones at five months of gestational age. She too experienced a delay in the attainment of gross motor skills, and attained head control by five months and sat independently at the age of 1.5 years. She began walking at the age of two, and her cognition was normal. She was operated for malunited subtrochanteric right sided foot at 1.5 years of age. At 5 years of age, she underwent corrective surgery for deformity in right thigh. Customised boots were provided during follow up. Her echocardiogram was unremarkable.

At 11 years of age, she measured 118 cm in height (-3.6 SD), 25 kg in weight (-2.18 SD), and had a head circumference of 50.5 cm (-1.71 SD). Her clinical features resembled her similarly affected sibling including short stature, bitemporal narrowing with posteriorly placed ears, micrognathia, microstomia, retrognathia, cleft uvula, crowded teeth, bilateral muscle wasting, and limited extension of the elbow, genu valgum and broad great toes (supplementary figure 1). Radiographs revealed radial head dislocation in right elbow joint, bent radius and ulna, mild scoliosis with platyspondyly, a broad, short pelvis with horizontal acetabulum, acute bending of femur, bowed tibia and fibula, bilateral wide femoral epiphyses with widening, and irregularities of epiphyses and metaphyses of the knee (figure 2-3, supplementary figure 2-6).

Family 2: A consanguineous (third degree) family had four pregnancies that included proband 3 (P3) with a kyphomelic dysplasia. The couple had an earlier medical abortion of a similarly affected fetus and have two healthy children (figure 1).

P3 was assessed at age 3.5 years and eleven-years. She was delivered at full term via lower segment caesarean section due to oligohydramnios. Her birth weight was 2.6 kg (-1.57 SD). Her motor development was delayed. She achieved head control at 4 months and independent sitting at eight months. She was not able to walk independently due to the severe skeletal deformities. She was able to stand with support on her toes at one year of age. She underwent surgery for cleft palate and posteromedial soft tissue release for clubfoot deformities at 9 months of age. She was first seen by us at the age of 3.5 years when she visited us for inability to walk, short stature and limbs deformities.

At 3.5 years, her height was 76 cm (-5.5 SD) and weight was 14 kg (+0.06 SD). At 11 years, her head circumference was 49 cm (-2.94 SD). The child exhibited short stature and facial dysmorphism that included bitemporal narrowing, posteriorly placed ears, a deviated nasal septum, microstomia, micrognathia, and retrognathia (Supplementary figure 1). Examination of the spine, chest, and abdomen revealed no abnormalities. Bilateral upper limb examination indicated normal shoulders, wrists, and hands; however, elbow varus and a 20° fixed flexion deformity were observed, with further flexion to 140°. Lower limb demonstrated normal hips, whereas knee joints exhibited 90° fixed flexion contractures, with further flexion up to 130°. Additionally, the patella and quadriceps mechanism were laterally dislocated. The feet showed calcaneovalgus deformity with dorsiflexion up to 30° and no plantar flexion.

Radiological evaluation revealed radial head dislocation, bent radius and ulna, bilateral patella joint dislocation and curved tibia and fibulae (figure 2-3, supplementary figure 2-6). She underwent corrective surgery for the knee deformity, which involved centralization of the patella and quadriceps mechanism through proximal and distal lateral releases and medial plication. Echocardiogram revealed a normal heart.

**References**

1. McKenna A, Hanna M, Banks E, Sivachenko A, Cibulskis K, Kernytsky A, et al. The Genome Analysis Toolkit: a MapReduce framework for analyzing next-generation DNA sequencing data. Genome Res. 2010 Sep;20(9):1297–303.

2. Wang K, Li M, Hakonarson H. ANNOVAR: functional annotation of genetic variants from high-throughput sequencing data. Nucleic Acids Res. 2010 Sep;38(16):e164.

3. Kausthubham N, Shukla A, Gupta N, Bhavani GS, Kulshrestha S, Das Bhowmik A, et al. A data set of variants derived from 1455 clinical and research exomes is efficient in variant prioritization for early-onset monogenic disorders in Indians. Hum Mutat. 2021 Apr;42(4):e15–61.

4. Sievers F, Wilm A, Dineen D, Gibson TJ, Karplus K, Li W, et al. Fast, scalable generation of high-quality protein multiple sequence alignments using Clustal Omega. Mol Syst Biol. 2011 Oct 11;7:539.

5. White RJ, Collins JE, Sealy IM, Wali N, Dooley CM, Digby Z, et al. A high-resolution mRNA expression time course of embryonic development in zebrafish. eLife. 6:e30860.

6. Wu RS, Lam II, Clay H, Duong DN, Deo RC, Coughlin SR. A Rapid Method for Directed Gene Knockout for Screening in G0 Zebrafish. Dev Cell. 2018 Jul 2;46(1):112-125.e4.

7. Medishetti R, Balamurugan K, Yadavalli K, Rani R, Sevilimedu A, Challa AK, et al. CRISPR-Cas9-induced gene knockout in zebrafish. STAR Protoc. 2022 Oct 26;3(4):101779.

8. Sorlien EL, Witucki MA, Ogas J. Efficient Production and Identification of CRISPR/Cas9-generated Gene Knockouts in the Model System Danio rerio. J Vis Exp. 2018 Aug 28;(138):56969.

9. Kimmel CB, Ballard WW, Kimmel SR, Ullmann B, Schilling TF. Stages of embryonic development of the zebrafish. Dev Dyn. 1995 Jul;203(3):253–310.

10. Walker MB, Kimmel CB. A two-color acid-free cartilage and bone stain for zebrafish larvae. Biotech Histochem. 2007 Feb;82(1):23–8.
