# Supplementary material for: Biallelic variants in CCN2 underlie an autosomal recessive kyphomelic dysplasia
Source: Eur J Hum Genet. 2024 Nov 6;33(1):30–7. doi: 10.1038/s41431-024-01725-5 (PMC11711675; doi:10.1038/s41431-024-01725-5)
Supplement: Supplementary file 2 — Supplementary material [file 41431_2024_1725_MOESM2_ESM.pptx]

## Slide 1
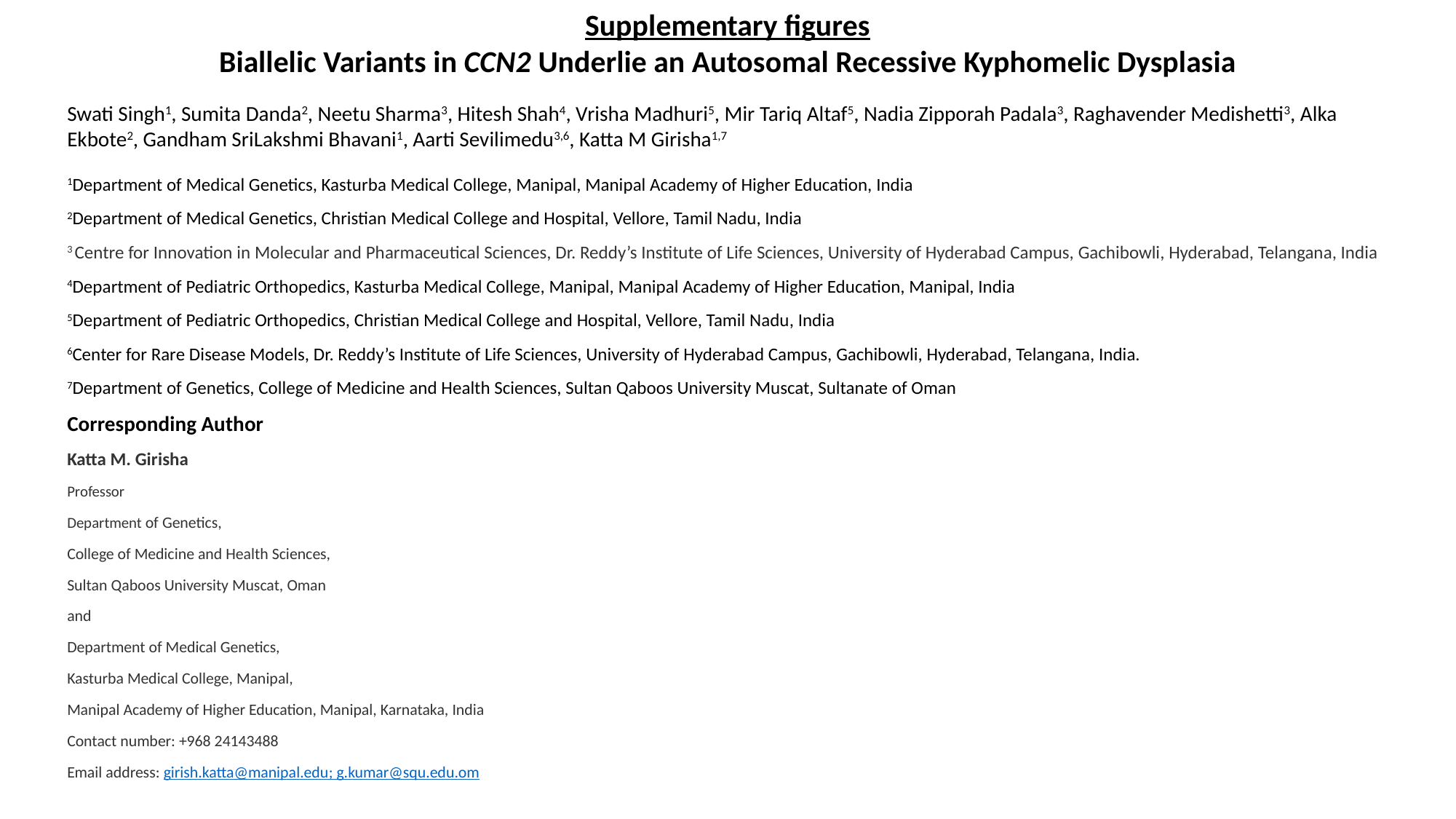

Supplementary figures
Biallelic Variants in CCN2 Underlie an Autosomal Recessive Kyphomelic Dysplasia
Swati Singh1, Sumita Danda2, Neetu Sharma3, Hitesh Shah4, Vrisha Madhuri5, Mir Tariq Altaf5, Nadia Zipporah Padala3, Raghavender Medishetti3, Alka Ekbote2, Gandham SriLakshmi Bhavani1, Aarti Sevilimedu3,6, Katta M Girisha1,7
1Department of Medical Genetics, Kasturba Medical College, Manipal, Manipal Academy of Higher Education, India
2Department of Medical Genetics, Christian Medical College and Hospital, Vellore, Tamil Nadu, India
3 Centre for Innovation in Molecular and Pharmaceutical Sciences, Dr. Reddy’s Institute of Life Sciences, University of Hyderabad Campus, Gachibowli, Hyderabad, Telangana, India
4Department of Pediatric Orthopedics, Kasturba Medical College, Manipal, Manipal Academy of Higher Education, Manipal, India
5Department of Pediatric Orthopedics, Christian Medical College and Hospital, Vellore, Tamil Nadu, India
6Center for Rare Disease Models, Dr. Reddy’s Institute of Life Sciences, University of Hyderabad Campus, Gachibowli, Hyderabad, Telangana, India.
7Department of Genetics, College of Medicine and Health Sciences, Sultan Qaboos University Muscat, Sultanate of Oman
Corresponding Author
Katta M. Girisha
Professor
Department of Genetics,
College of Medicine and Health Sciences,
Sultan Qaboos University Muscat, Oman
and
Department of Medical Genetics,
Kasturba Medical College, Manipal,
Manipal Academy of Higher Education, Manipal, Karnataka, India
Contact number: +968 24143488
Email address: girish.katta@manipal.edu; g.kumar@squ.edu.om

## Slide 2
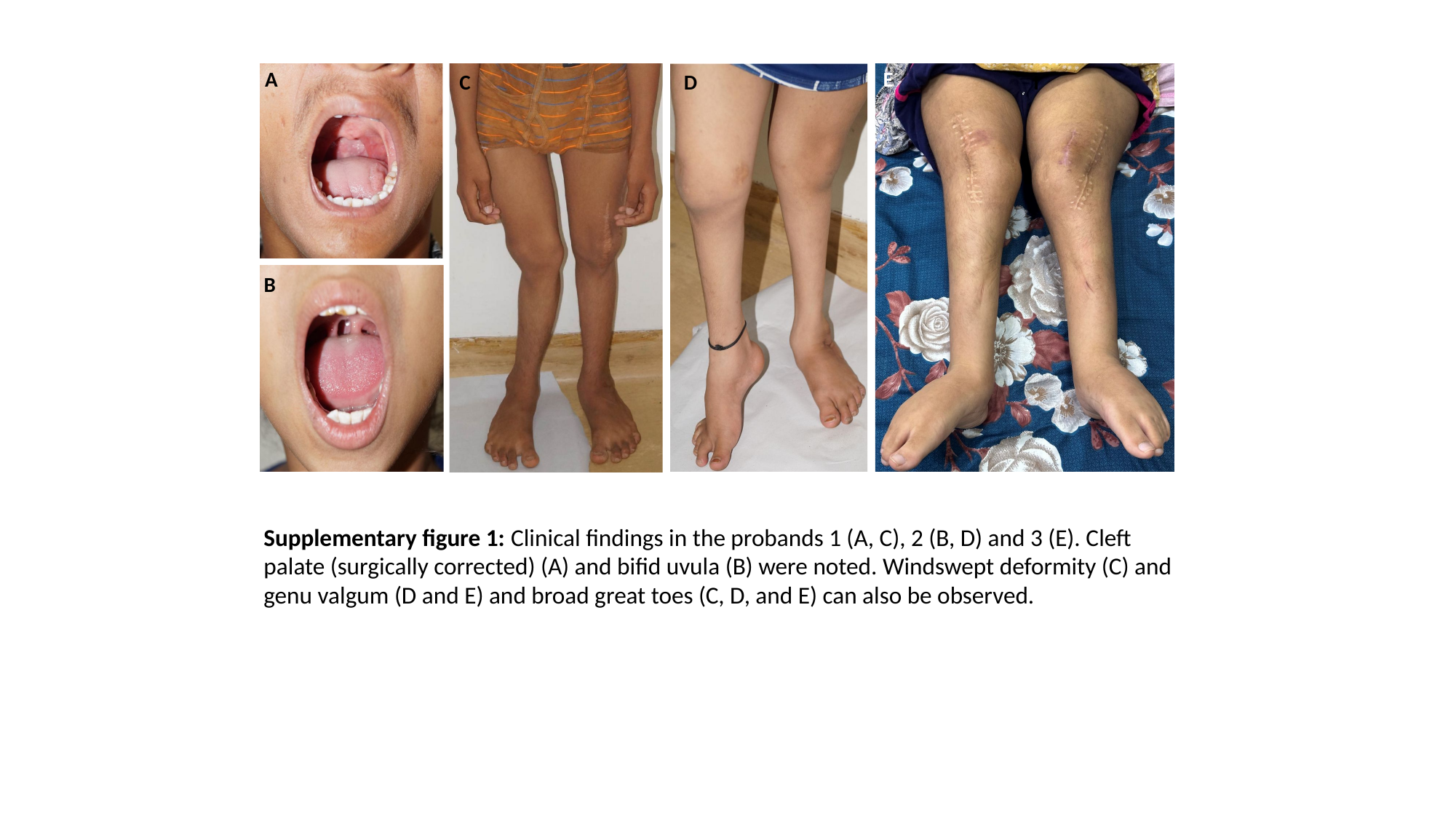

E
A
B
C
D
I
K
Supplementary figure 1: Clinical findings in the probands 1 (A, C), 2 (B, D) and 3 (E). Cleft palate (surgically corrected) (A) and bifid uvula (B) were noted. Windswept deformity (C) and genu valgum (D and E) and broad great toes (C, D, and E) can also be observed.

## Slide 3
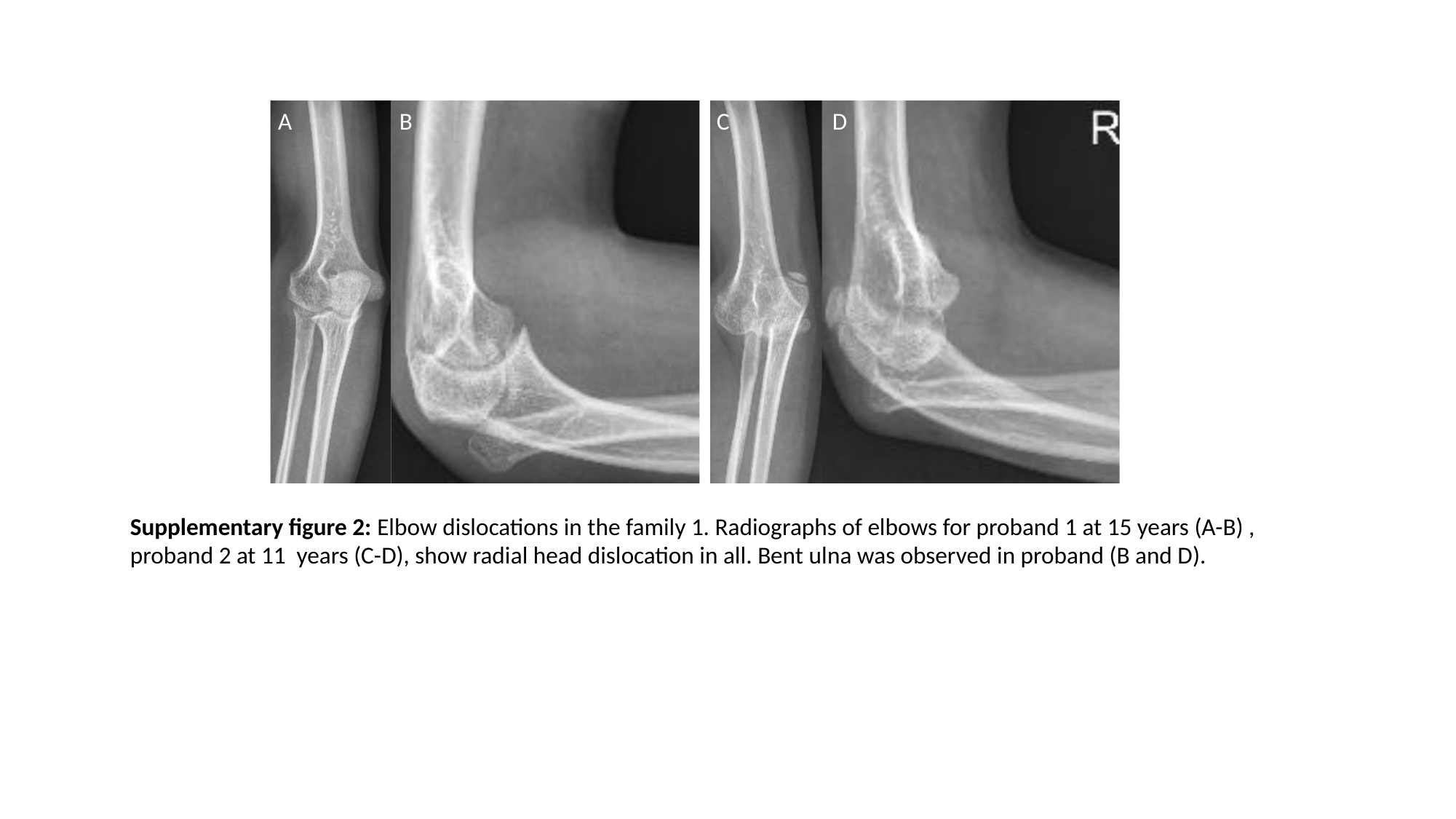

#
B
A
C
D
Supplementary figure 2: Elbow dislocations in the family 1. Radiographs of elbows for proband 1 at 15 years (A-B) , proband 2 at 11 years (C-D), show radial head dislocation in all. Bent ulna was observed in proband (B and D).

## Slide 4
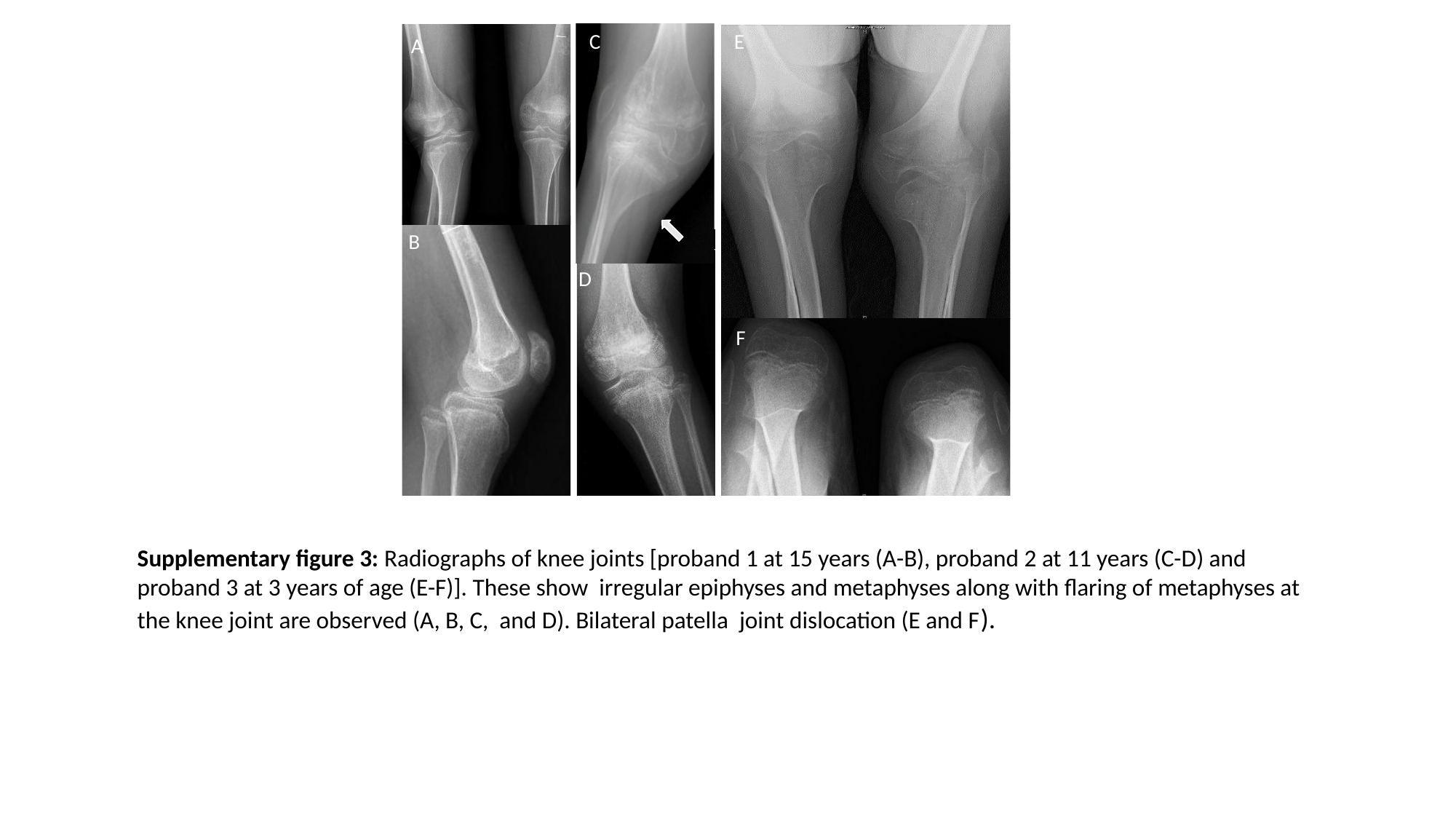

B
C
H
E
A
J
B
F
D
F
A
Supplementary figure 3: Radiographs of knee joints [proband 1 at 15 years (A-B), proband 2 at 11 years (C-D) and proband 3 at 3 years of age (E-F)]. These show irregular epiphyses and metaphyses along with flaring of metaphyses at the knee joint are observed (A, B, C, and D). Bilateral patella joint dislocation (E and F).

## Slide 5
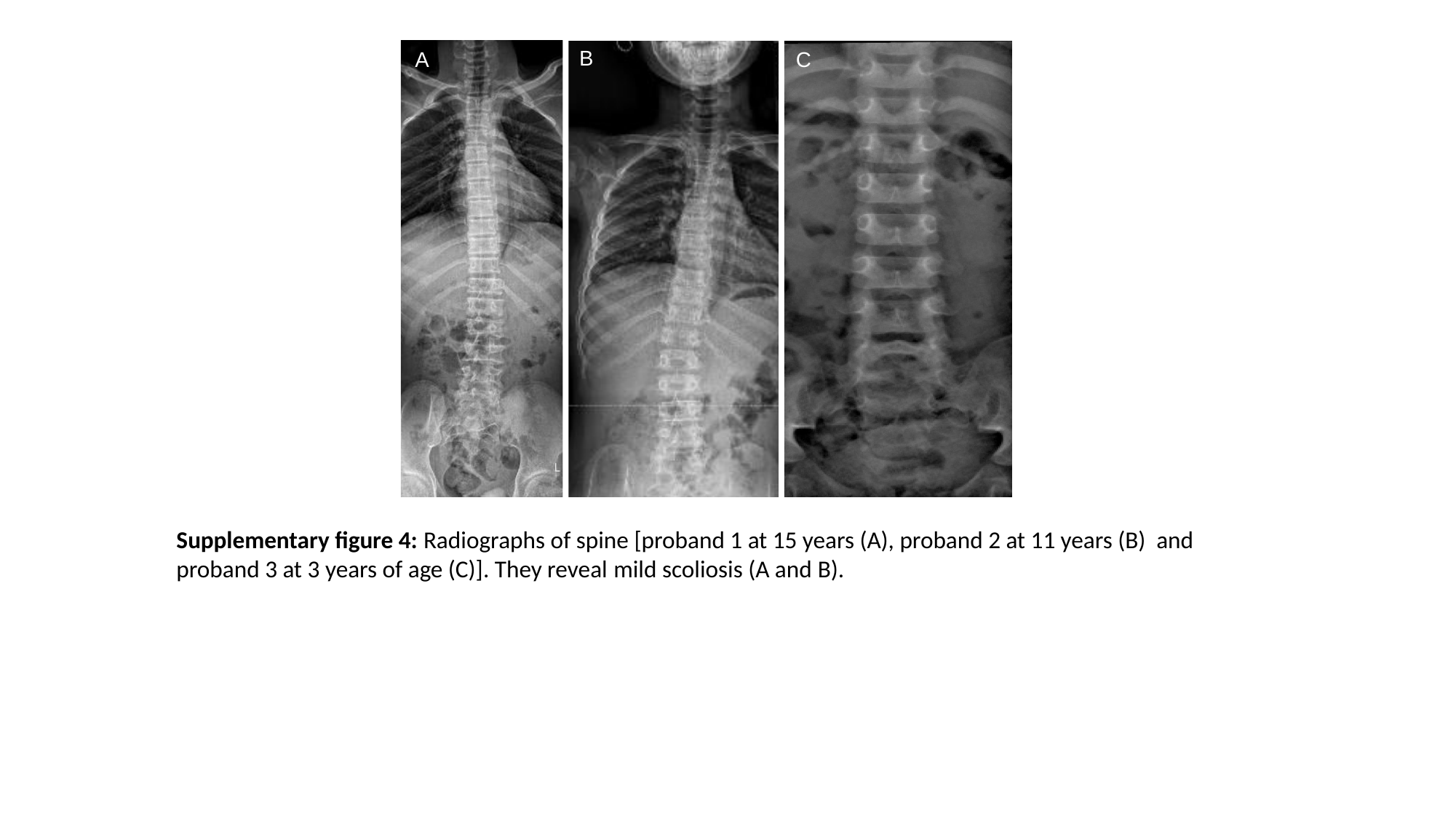

F
C
B
D
B
A
Supplementary figure 4: Radiographs of spine [proband 1 at 15 years (A), proband 2 at 11 years (B) and proband 3 at 3 years of age (C)]. They reveal mild scoliosis (A and B).

## Slide 6
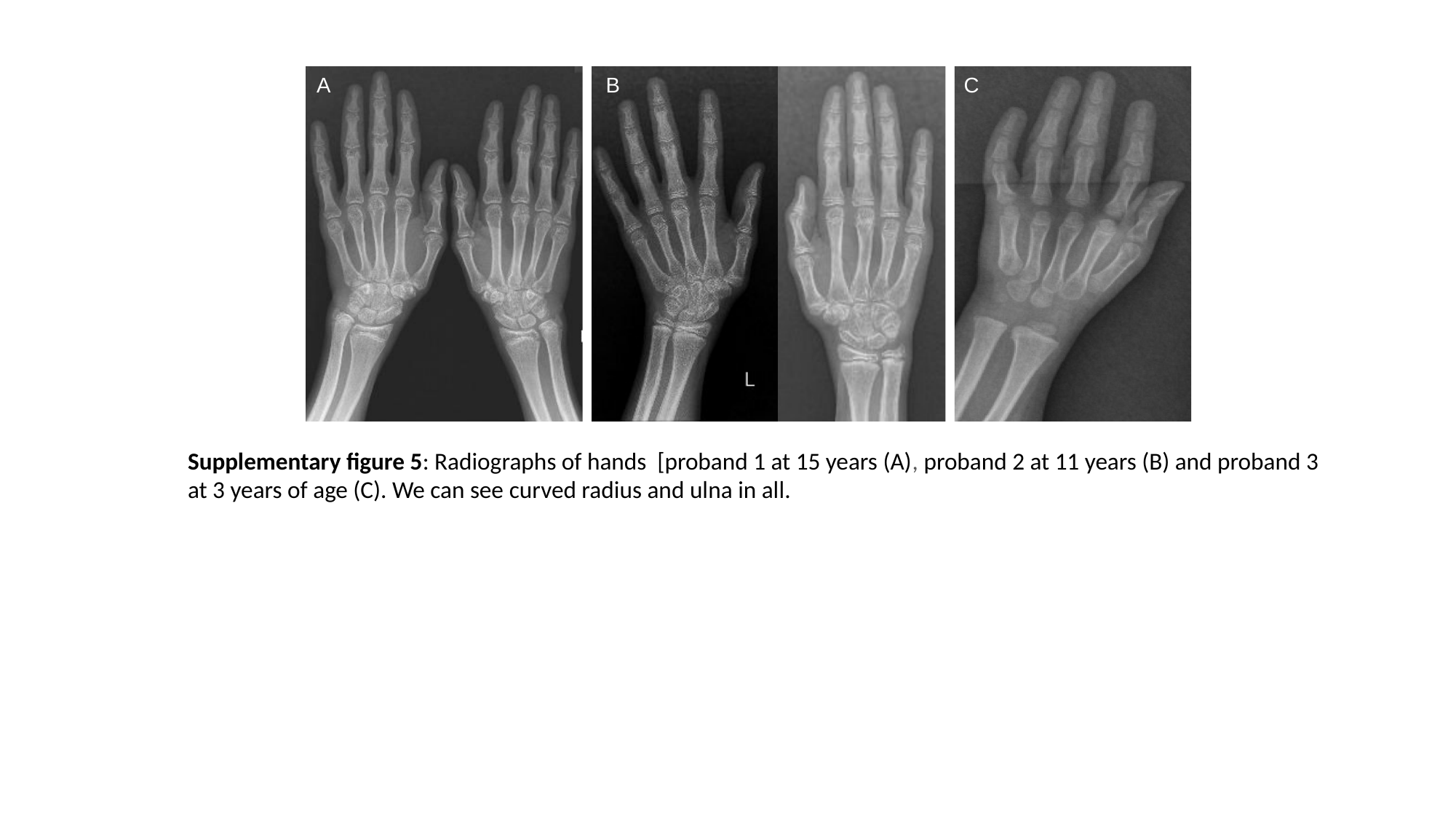

C
A
B
Supplementary figure 5: Radiographs of hands [proband 1 at 15 years (A), proband 2 at 11 years (B) and proband 3 at 3 years of age (C). We can see curved radius and ulna in all.

## Slide 7
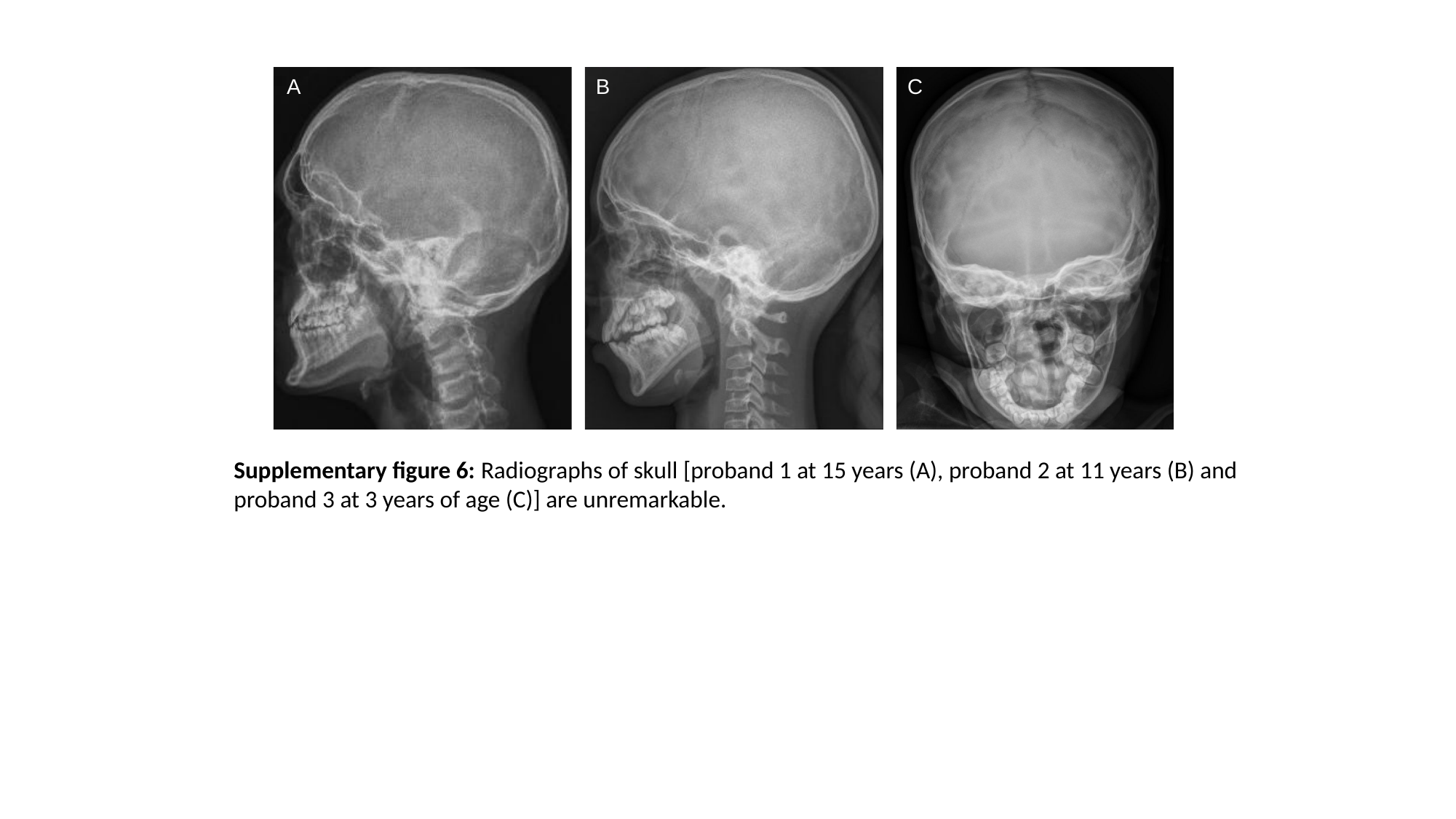

A
A
B
C
Supplementary figure 6: Radiographs of skull [proband 1 at 15 years (A), proband 2 at 11 years (B) and proband 3 at 3 years of age (C)] are unremarkable.

## Slide 8
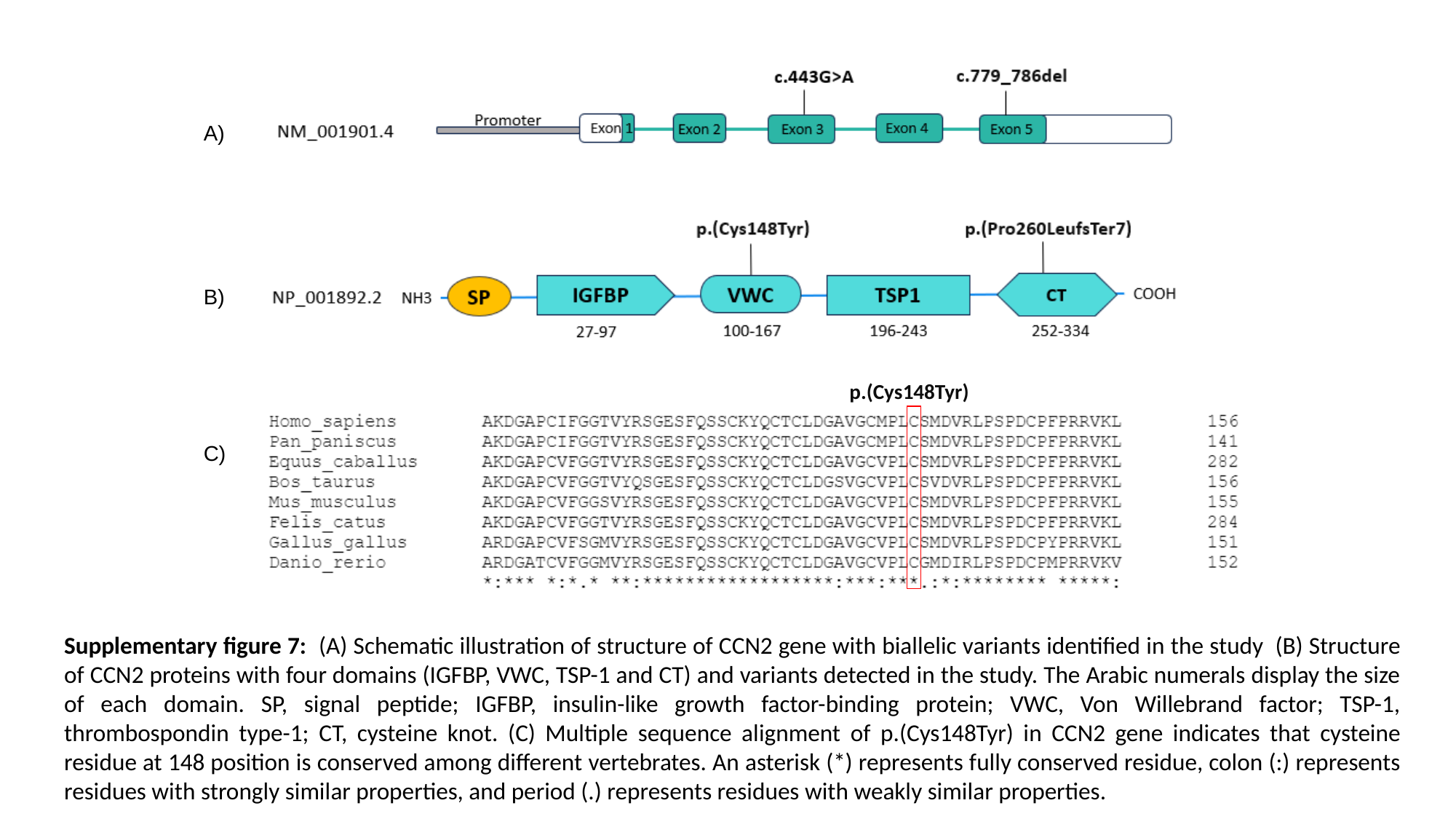

A)
B)
p.(Cys148Tyr)
C)
Supplementary figure 7: (A) Schematic illustration of structure of CCN2 gene with biallelic variants identified in the study (B) Structure of CCN2 proteins with four domains (IGFBP, VWC, TSP-1 and CT) and variants detected in the study. The Arabic numerals display the size of each domain. SP, signal peptide; IGFBP, insulin-like growth factor-binding protein; VWC, Von Willebrand factor; TSP-1, thrombospondin type-1; CT, cysteine knot. (C) Multiple sequence alignment of p.(Cys148Tyr) in CCN2 gene indicates that cysteine residue at 148 position is conserved among different vertebrates. An asterisk (*) represents fully conserved residue, colon (:) represents residues with strongly similar properties, and period (.) represents residues with weakly similar properties.

## Slide 9
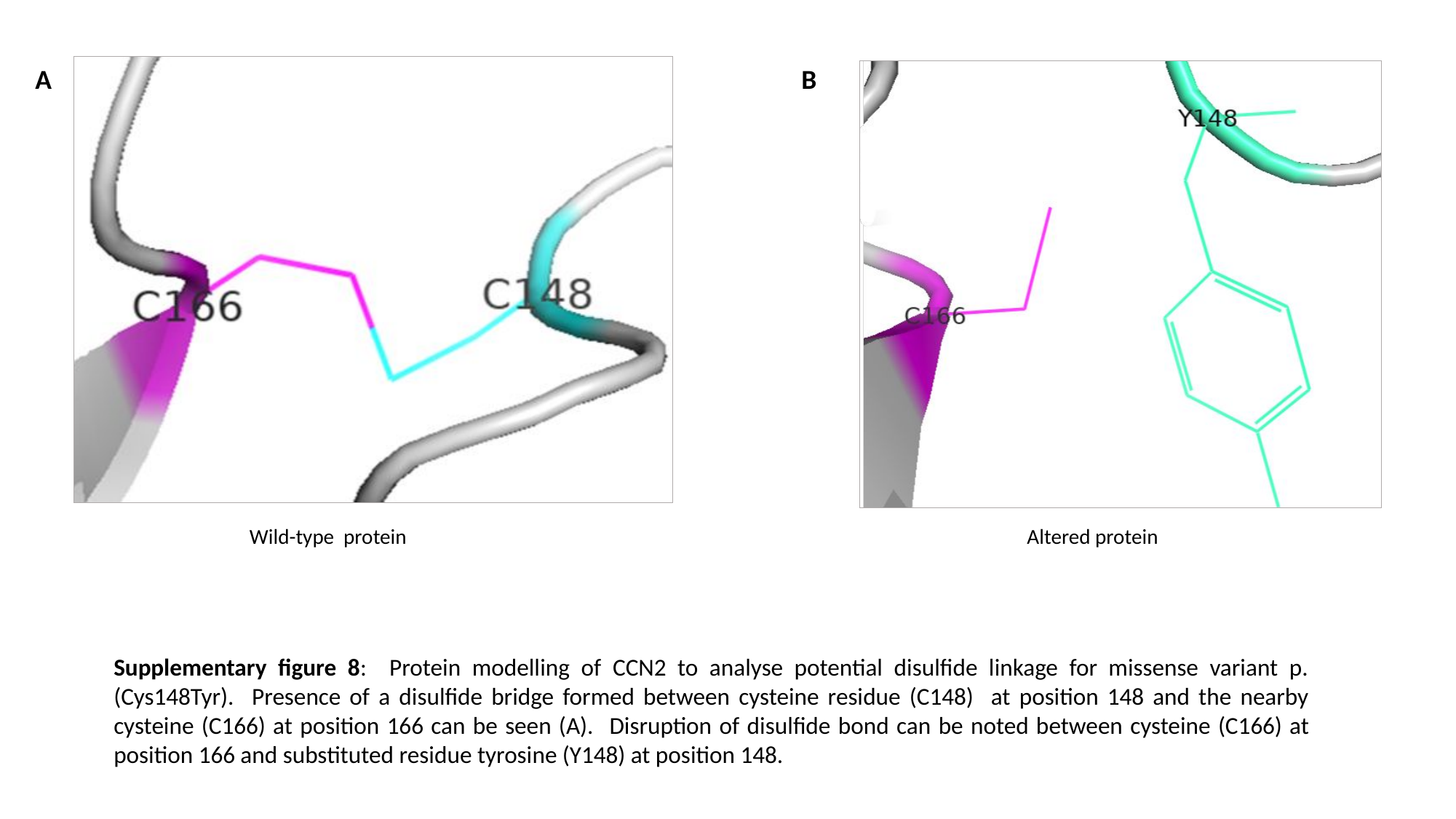

A
B
Wild-type protein
Altered protein
Supplementary figure 8: Protein modelling of CCN2 to analyse potential disulfide linkage for missense variant p.(Cys148Tyr). Presence of a disulfide bridge formed between cysteine residue (C148) at position 148 and the nearby cysteine (C166) at position 166 can be seen (A). Disruption of disulfide bond can be noted between cysteine (C166) at position 166 and substituted residue tyrosine (Y148) at position 148.

## Slide 10
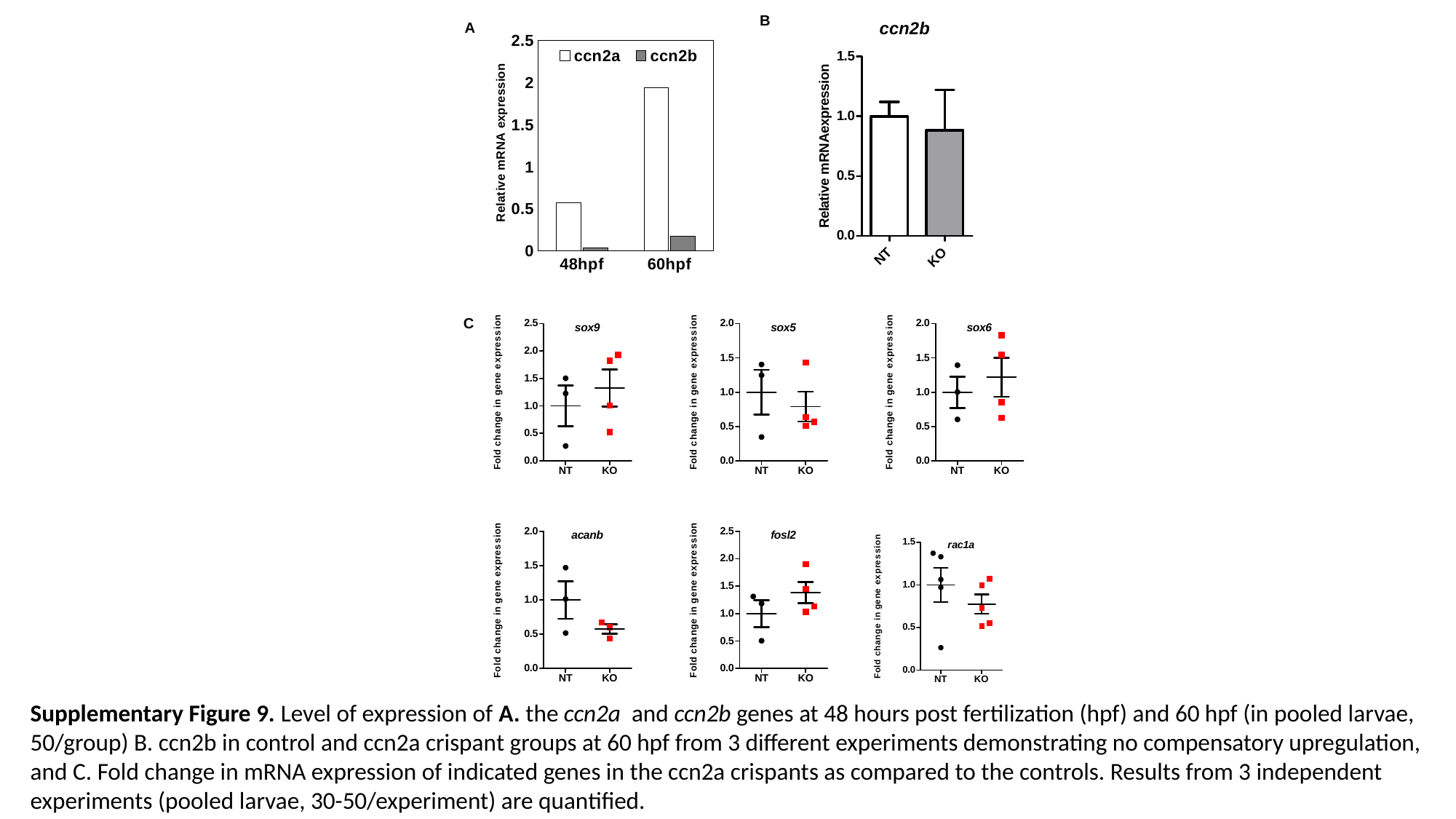

B
A
### Chart
| Category | ccn2a | ccn2b |
|---|---|---|
| 48hpf | 0.5761324974656178 | 0.03292169763248173 |
| 60hpf | 1.9422953687700357 | 0.16982127571047276 |
C
Supplementary Figure 9. Level of expression of A. the ccn2a and ccn2b genes at 48 hours post fertilization (hpf) and 60 hpf (in pooled larvae, 50/group) B. ccn2b in control and ccn2a crispant groups at 60 hpf from 3 different experiments demonstrating no compensatory upregulation, and C. Fold change in mRNA expression of indicated genes in the ccn2a crispants as compared to the controls. Results from 3 independent experiments (pooled larvae, 30-50/experiment) are quantified.

## Slide 11
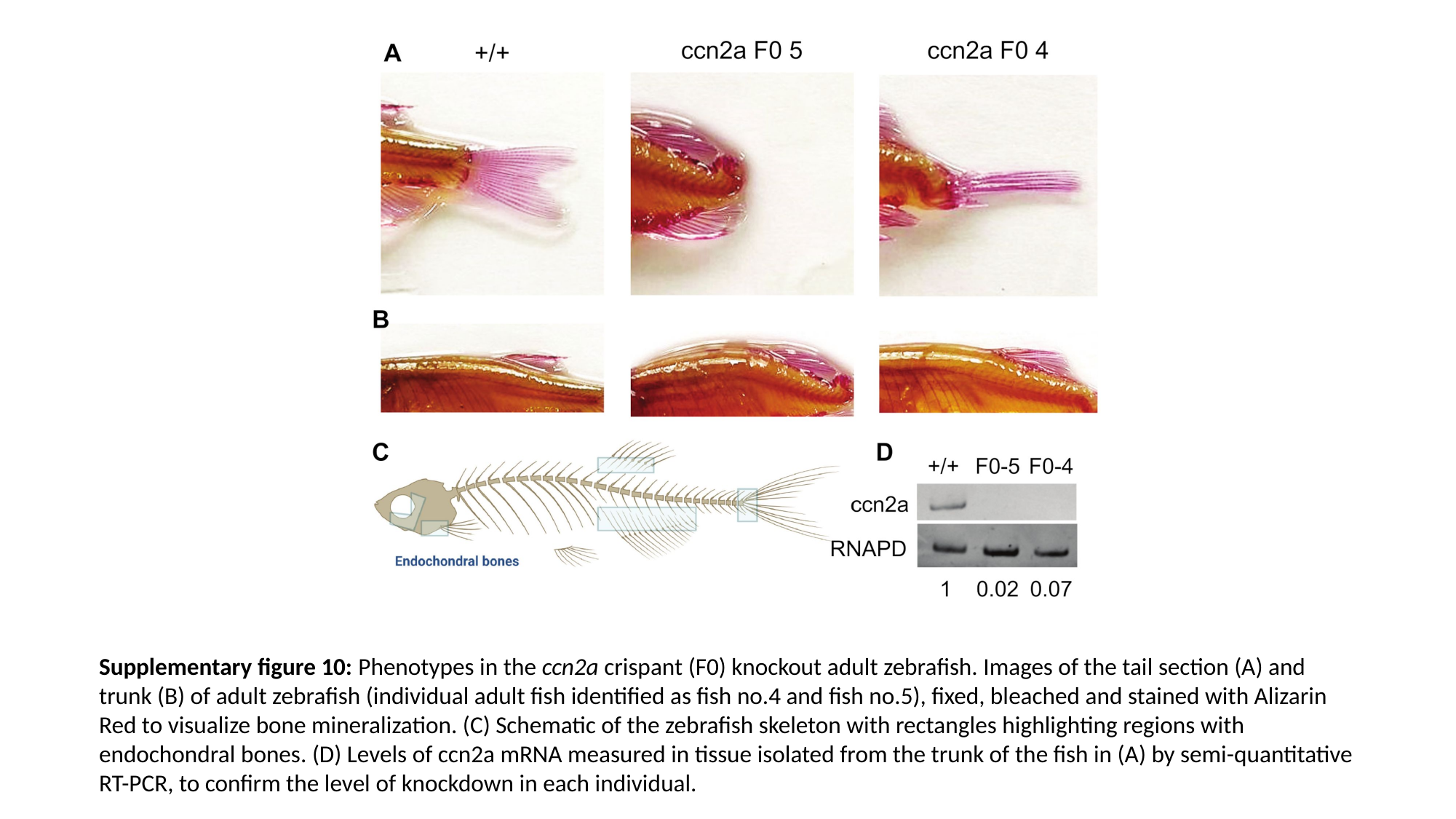

Supplementary figure 10: Phenotypes in the ccn2a crispant (F0) knockout adult zebrafish. Images of the tail section (A) and trunk (B) of adult zebrafish (individual adult fish identified as fish no.4 and fish no.5), fixed, bleached and stained with Alizarin Red to visualize bone mineralization. (C) Schematic of the zebrafish skeleton with rectangles highlighting regions with endochondral bones. (D) Levels of ccn2a mRNA measured in tissue isolated from the trunk of the fish in (A) by semi-quantitative RT-PCR, to confirm the level of knockdown in each individual.

## Slide 12
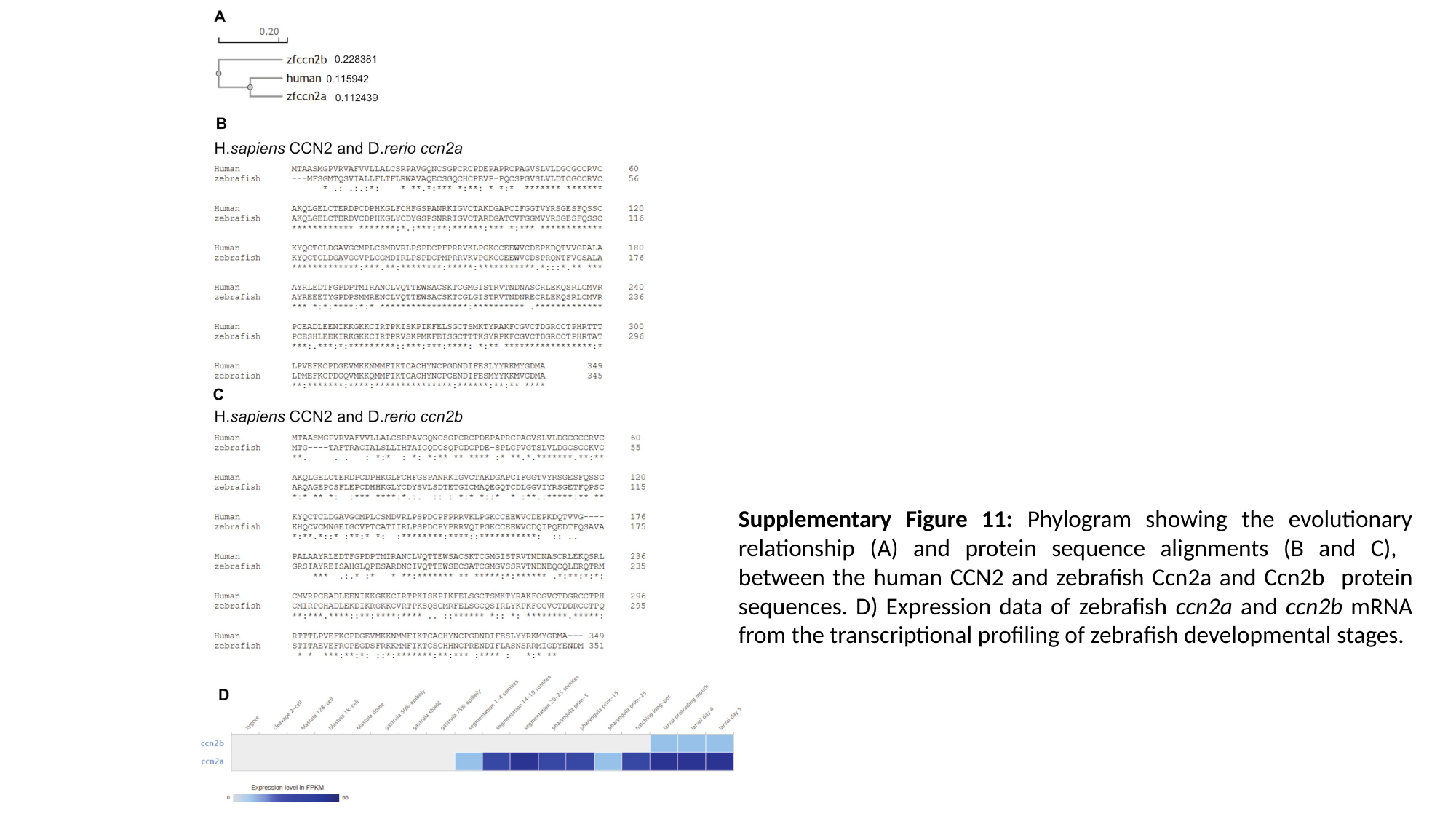

Supplementary Figure 11: Phylogram showing the evolutionary relationship (A) and protein sequence alignments (B and C), between the human CCN2 and zebrafish Ccn2a and Ccn2b protein sequences. D) Expression data of zebrafish ccn2a and ccn2b mRNA from the transcriptional profiling of zebrafish developmental stages.

## Slide 13
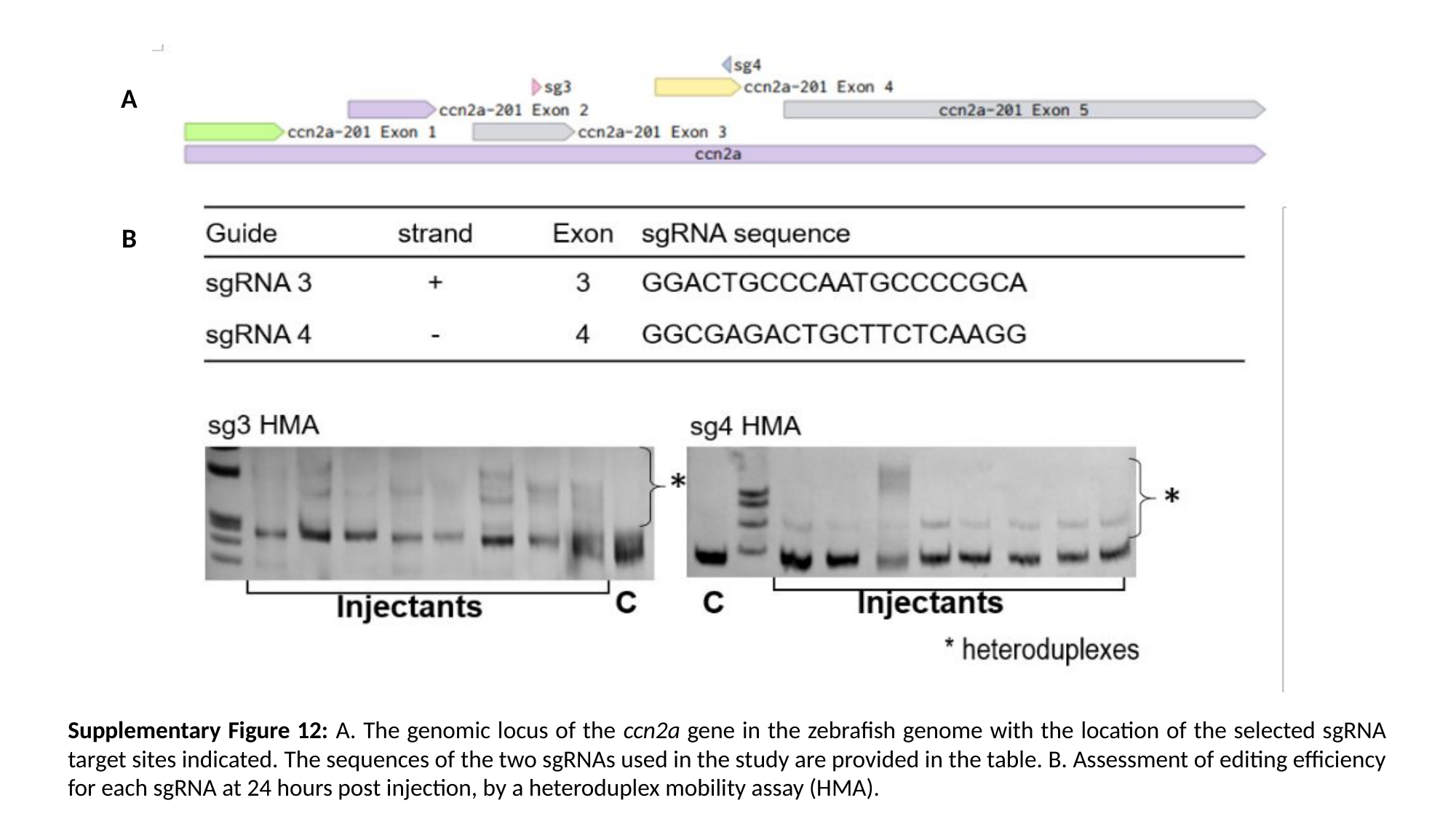

A
B
Supplementary Figure 12: A. The genomic locus of the ccn2a gene in the zebrafish genome with the location of the selected sgRNA target sites indicated. The sequences of the two sgRNAs used in the study are provided in the table. B. Assessment of editing efficiency for each sgRNA at 24 hours post injection, by a heteroduplex mobility assay (HMA).
